# Application of bivariate mixed counting process models to genetic analysis of rheumatoid arthritis severity

**DOI:** 10.1186/1753-6561-1-s1-s120

**Published:** 2007-12-18

**Authors:** Rinku Sutradhar, Dushanthi Pinnaduwage, Shelley B Bull

**Affiliations:** 1 Samuel Lunenfeld Research Institute of Mount Sinai Hospital, 60 Murray Street, Box #18, Lebovic Building, 5^th ^Floor, Prosserman Centre, Toronto, Ontario M5T 3L9, Canada; 2 Department of Public Health Sciences, Faculty of Medicine, University of Toronto, Health Sciences Building, 6^th ^Floor, 155 College Street, Toronto, Ontario MST 3M7, Canada

## Abstract

We sought to i) identify putative genetic determinants of the severity of rheumatoid arthritis in the NARAC (North American Rheumatoid Arthritis Consortium) data, ii) assess whether known candidate genes for disease status are also associated with disease severity in those affected, and iii) determine whether heterogeneity among the severity phenotypes can be explained by genetic and/or host factors. These questions are addressed by developing bivariate mixed-counting process models for numbers of tender and swollen joints to evaluate genetic association of candidate polymorphisms, such as *DRB1*, and selected single-nucleotide polymorphisms in known candidate genes/regions for rheumatoid arthritis, including *PTPN22*, and those in the regions identified by a genome-wide linkage scan of disease severity using the dense Illumina single-nucleotide polymorphism panel. The counting process framework provides a flexible approach to account for the duration of rheumatoid arthritis, an attractive feature when modeling severity of a disease. Moreover, we found a gain in efficiency when using a bivariate compared to a univariate counting process model.

## Background

The NARAC (North American Rheumatoid Arthritis Consortium) data provided for Genetic Analysis Workshop 15 (GAW15) includes 757 families with 8017 individuals representing multiple ethnicities. The data include information on family relationships, discrete and quantitative phenotypes, covariates, and genome-wide microsatellite genotypes and single-nucleotide polymorphism (SNP) genotypes from Illumina as well as genetic locations for microsatellites and physical locations for SNPs [1].

Our data set for association analysis consists of information on 1492 individuals in 710 families selected from the NAPHENO data. Subjects were included only if information on "TenderJtCt", "SwollenJtCt", "YrOnset", and "YrAscer" are available. The severity phenotypes include the joint count variables "TenderJtCt" and "SwollenJtCt". When examined with respect to the duration of rheumatoid arthritis (RA) (calculated by subtracting "YrOnset" from "YrAscer"), the joint variables can be viewed as a bivariate counting process. Note that the observed time since RA onset varied from 1 to 72 years, and the number of joints affected ranged from 0 to 28 and 0 to 26 for tender and swollen, respectively.

Our analytic strategy consisted of the following steps. We began by performing a genome-wide linkage scan using the Illumina SNP panel to identify significant regions of linkage for each count variable. Much of our attention focused on chromosome 6, where a significant region of linkage (harboring the *HLA-DRB1 *locus) has been previously reported for RA (disease status) [1]. The genome-wide linkage analysis not only indicated regions that may be fine-mapped via association analysis, but also suggested differences in linkage signals between the count phenotypes. This motivated the formulation of the bivariate mixed-counting process framework that jointly modeled both tender and swollen processes, as well as detecting differences in patterns of association. We were particularly interested in evaluating the genetic association of candidate polymorphisms, such as *DRB1*, as well as SNPs selected by genome-wide linkage analysis, or within known candidate genes such as *PTPN22*, previously reported to be associated with an increased risk of RA [2]. Due to restrictions on available software for linkage analysis, the phenotypes were not modeled in the same manner as for association analysis; the purpose of the linkage analysis was simply to provide a preliminary understanding of the linkage behavior and potential differences between the tender and swollen joint count phenotypes.

### Genome-wide linkage analysis with SNPs

A genome-wide linkage scan of the count variables available in 1519 individuals in 710 families using 5744 SNPs (Illumina panel) was performed via MERLIN-REGRESS (version 1.0.1) [3], with the square root transformation of the counts treated as quantitative traits. In this regression-based method [4], we used the sample estimates of the transformed counts as population means and variances, and estimated heritability using the variance-components (VC) option in MERLIN (version 1.0.1) [3], with values of 39% and 42% for transformed swollen and tender joint counts, respectively. Because genetic maps were not available for the SNPs, we assumed that 1 Mb is equivalent to 1 cM.

**Figure 1:**
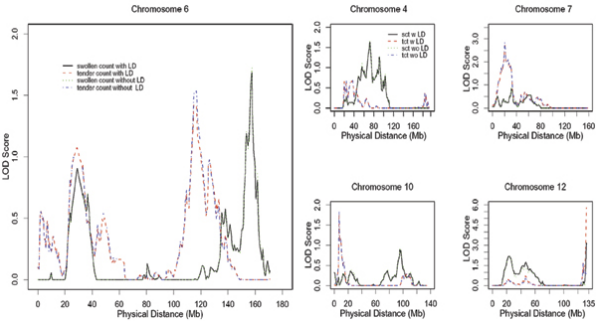
LOD score results from genome-wide linkage analysis.

Directing our attention to chromosome 6, which has been previously reported to show a significant region of linkage (harboring the *HLA-DRB1 *locus) for RA, we found two regions of modest signal for each trait (see Fig. 1). One region detected for both count variables spanned 26 to 33 Mb. A second region for the tender count variable spanned 114 to 117 Mb, and a differing secondary region for the swollen count variable spanned 155 to 158 Mb. Several other regions of interest were also detected on other chromosomes (Fig. 1). Adjusting this multipoint analysis for linkage disequilibrium using MERLIN produced minor changes (Fig. 1).

## Methods

### A bivariate counting process model for genetic association of RA severity

Methods for analyzing data on events observed over time have been of considerable interest in recent years. A counting process framework [5,6] models count data, collected at fixed points of ascertainment, by taking duration of disease into consideration (as is the case in a cross-sectional design, provided that the time of disease onset is available). Under the assumption that the numbers of tender and swollen joints do not decrease through time, we have a recurrent event process.

Let {N_*ij*_(t), t ≥ 0} represent the underlying counting process in which N_*ij*_(t) denotes the number of events experienced by the *j*^th ^process of the *i*^th ^individual over the continuous time interval (0, t], where *i *= 1,..., *n*. Note that *j *= 1, 2 represents the tender and swollen joint counting processes, respectively. Because it is reasonable to specify a model that depends only on the current covariate values rather than on the entire history process in our setting, we formulated a non-homogeneous Poisson process. To handle any substantial inter-individual variation in the model, we introduced a positive individual-specific random effect *u*_*i *_that is shared for both processes of the *i*^th ^subject. Conditional on *u*_*i*_, the counts of the *i*^th ^subject are independent. To be more specific, we assumed that conditional on *u*_*i*_, the counting process {N_*ij*_(t), t ≥ 0} is a non-homogeneous Poisson process with intensity and mean functions represented as

λ_*ij *_(t|*u*_*i*_) = *u*_*i*_λ_0*j*_(t) exp{**x**_*ij*_^T^**β**_*j*_}

and

Λ_*ij *_(t|*u*_*i*_) = *u*_*i*_Λ_0*j*_(t) exp{**x**_*ij*_^T^**β**_*j*_},

respectively. The time line is defined as the time since onset of RA. The form of the intensity function is a relative risk or Cox model, with the baseline intensity function for the *j*^th ^process λ_0*j*_(t) common among all subjects. The covariate vector **x**_*ij*_, specific to the *i*^th ^subject, may include candidate polymorphisms (such as *DRB1*), single SNPs selected through genome-wide linkage scans, or a vector of SNPs chosen to capture variation in a candidate gene or candidate region (such as *PTPN22*); it may also include characteristics such as sex and smoking history, or any combination of reasonable interactions. The corresponding regression parameter vector **β**_*j *_describes the association of genotype with phenotype for the *j*^th ^process. Furthermore, each individual has their own frailty *u*_*i *_acting multiplicatively on the intensity function.

Unless the variance of the underlying mixing distribution is exceptionally large, the gamma random effect provides a robust approach for modeling mixed-Poisson processes [7]. Assuming that *u*_1_,..., *u*_*n *_are independent and identically distributed random variables arising from a gamma distribution with mean of 1 and variance of φ, we obtain the expectation E[N_*ij*_(t)] = E (E[N_*ij*_(t)|*u*_*i*_]) = Λ_0*j*_(t) exp{**x**_*ij*_^T^**β**_*j*_}, which represents the mean number of counts for the *j*^th ^process of the *i*^th ^individual over the interval (0, t]. The parameter φ is a measure of heterogeneity between individuals that may not have been sufficiently accounted for by the Poisson model alone. Larger values of φ imply extra-Poisson variation in the model.

We now need to construct a likelihood function based on our bivariate mixed-Poisson process assumptions. Suppose the *i*^th ^individual is ascertained at time τ_*i*_, which is relative to the time of onset. Conditional on the random effect *u*_*i*_, the distribution of the counts P(N_*ij*_(τ_*i*_) = n_*ij*_|*u*_*i*_) has a Poisson form. Moreover, under conditional independence, the bivariate distribution of the counts can be computed as P(n_*i*1_, n_*i*2_) = ∫ P(n_*i*1_, n_*i*2_|*u*_*i*_) *d*G(*u*_*i*_) = ∫ P(n_*i*1_|*u*_*i*_) *P*(*n*_*i*2_|*u*_*i*_) *g*(*u*_*i*_) *du*_*i*_, which has a convenient closed form expression due to the gamma-Poisson mixture. Thus the log-likelihood log L(**θ**) = Σ log P(n_*i*1_, n_*i*2_) is maximized with respect to **θ**, which consists of parameters **β**_1_, **β**_2_, φ, and parameters of the baseline intensity functions. We applied a Newton-Raphson technique, but any non-linear maximization algorithm may be used for this purpose. Furthermore, when maximizing the log-likelihood under the counting process framework, it is most convenient to assume a parametric form such as a Weibull model for the baseline intensity functions, although semi-parametric assumptions using piecewise constant models are also reasonable alternatives.

In the case of a univariate counting process model, there is no joint distribution formulated among the counts for each individual. Rather, the log-likelihood is simply L(**θ**) = ΣΣ log P(n_*ij*_), where P(n_*ij*_) = ∫ P(n_*ij*_|*u*_*i*_) *d*G(*u*_*i*_) = ∫ P(n_*ij*_|*u*_*i*_) *g*(*u*_*i*_) *du*_*i*_.

## Results and discussion

### Association analysis of candidate gene *DRB1*

**Figure 2:**
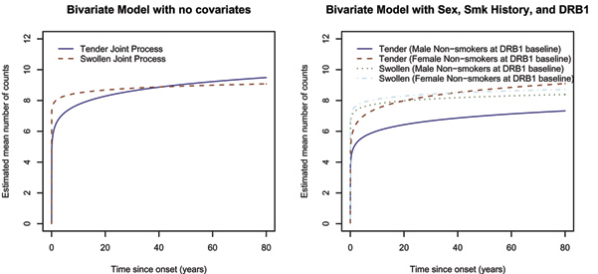
Estimated mean number of counts versus time since RA onset.

We applied our bivariate mixed-counting process model for the severity phenotypes under various covariate models, yielding estimates and standard errors of **β**_1_, **β**_2_, and log φ (Table 1). The asymptotic confidence interval for log φ indicated a significant amount of extra-Poisson variation in the counts. That is, the number of tender joints varied considerably between patients, as did the number of swollen joints. The heterogeneity appeared to decrease as more covariates were added to the model, and any remaining variation in the model was captured under this random effects formulation. The log-likelihood increased dramatically as the covariates sex, smoking history, and *DRB1 *were added to the model. The likelihood ratio (LR) test for the joint contribution of sex to the bivariate model (2 degrees of freedom) provided a *p*-value less than 1 × 10^-6^, and the LR test for the joint contribution of *DRB1 *provided a *p*-value less than 0.0001. The Wald test detected a significant sex effect for the tender, but not for the swollen joint process. These sex differences were evident in plots of the estimated mean number of counts (Fig. 2). The LR test for the equality of DRB1 between the two counting processes in the bivariate model (1 degree of freedom) yielded a *p*-value less than 1 × 10^-5^.

**Table 1:** Results under a bivariate mixed-counting process model for genetic association of RA severity

				Covariate associations		
				
Model	log L	Heterogeneity log φ (SE)	Outcome	Sex β (SE)	Smoking history β (SE)	*DRB1 *β (SE)
No covariates	-10052.06	-0.358 (0.04)	Tender			
			Swollen			
With sex	-10035.23	-0.360 (0.04)	Tender	-0.185 (0.05)		
			Swollen	-0.010 (0.05)		
With sex,	-10031.06	-0.365 (0.04)	Tender	-0.216 (0.06)	0.139 (0.04)	
smoking history			Swollen	-0.035 (0.05)	0.110 (0.04)	
With sex,	-10020.33	-0.366 (0.04)	Tender	-0.216 (0.06)	0.139 (0.05)	-0.009 (0.03)
smoking history, and *DRB1*			Swollen	-0.038 (0.06)	0.107 (0.05)	0.071 (0.03)

Relative efficiency computations (not shown), obtained by taking the ratio of the variances, suggested a gain in information for the bivariate versus univariate counting process model. For the tender and swollen outcomes, the relative variance ranged from 1.122 to 1.294 and 1.005 to 1.015, respectively, under various covariates.

### Association analysis of the 14 PTPN22 SNPs on chromosome 1 and 404 illumina SNPs on chromosome 6

To evaluate the genetic association of candidate gene *PTPN22 *and SNPs on chromosome 6, we performed various LR tests under both univariate and bivariate mixed-counting process models. Under the univariate model we tested

(i) H_*o *_: β_*tender*, *SNP *_= 0 and (ii) H_*o*_: β_*swollen*, *SNP *_= 0,

and under the bivariate model we tested

(i) H_*o *_: β_*tender*, *SNP *_= 0, β_*swollen*, *SNP *_= 0 and (ii) H_*o *_: β_*tender*, *SNP *_= β_*swollen*, *SNP*_.

**Figure 3:**
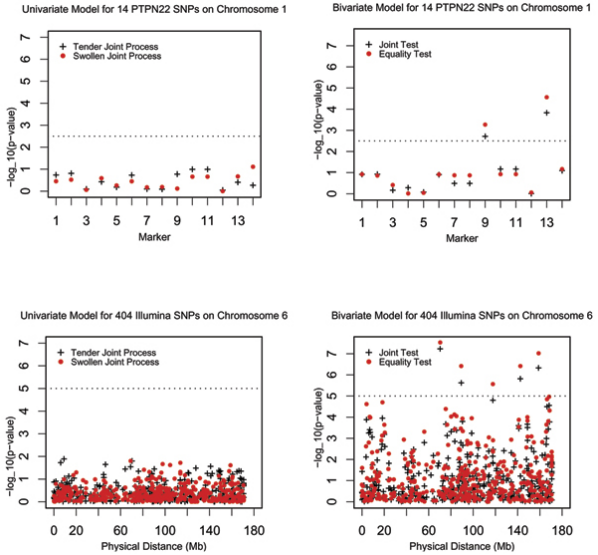
Likelihood ratio test *p*-values under univariate and bivariate models (with sex, smoking history, and *DRB1*) for SNPs on chromosome 1 and 6.

Note that, along with sex and smoking history, *DRB1 *was included in the model since it is significant based on the results of the association analysis above, and also because it has been previously reported to show strong linkage [1] and association with RA. Figure 3 consists of plots of the *p*-values for each SNP. The dashed line indicates a region-wide Bonferroni significance criterion, computed as the ratio of a selected significant *p*-value (0.05) over the number of tests performed.

## Conclusion

The counting process model examines phenotypes in a longitudinal framework by taking duration of disease into account. This model is applicable in a cross-sectional design, provided that the time of disease onset is available. Genetic association analysis under a bivariate model provides a stronger approach than treating each outcome separately. The use of the counting process to model RA severity is novel, so our primary focus is the association between the bivariate count phenotypes and main effects, however an extension to incorporate gene × gene and gene × environment interactions would be straightforward.

## Competing interests

The author(s) declare that they have no competing interests.
